# 

**Published:** 1916-02-19

**Authors:** 


### CONVALESCENT HOME FOR WESTMORLAND.

Mrs. Mary Elizabeth Williams, of Holme Island, Grange-on-Sands, Lancs, left such part of the Holme Island or the Jewbarrow estates as the trustees may consider suitable for the establishment of a convalescent home, to be called the "Barrow Thornborrow Convalescent Institution for the Benefit of Sick, Weak, or Infirm People of the County of Westmorland," and a block of houses, shops, and buildings on the Jewbarrow estate, to form an endowment fund. She also left £200 to the County Hospital at Kendal, £100 to the Meathop Sanatorium for Consumption, and the residue of her estate for other charitable objects.

### Memorial to Miss Katharine Monk.

ALL connected with King's College Hospital will recognise its great obligations to Miss Katharine Monk, for twenty-one years sister-matron of the hospital, whose death occurred on February 20, 1915. Miss Monk became sister-matron when the charge of the nursing of the hospital was relinquished by the St. John's Sisterhood. She was thus the founder of the Nursing School of King's College Hospital, and inspired its special characteristics. She was known as one of the most able of the many able women who have been at the head of the nursing departments of the great London hospitals. She gave her time, her health, and her strength, ungrudgingly to the service of King's College Hospital; and in particular she devoted all her special knowledge and experience to planning the new buildings at Denmark Hill. Her kindness and her capacity will be remembered by many generations of those who worked with her. It is felt that there will be many who will be anxious to contribute to some memorial to her, and a committee has been formed to carry out this purpose. It is proposed to erect some memorial in the chapel, and, if funds permit, to form an endowment in connection with the Nurses' Home or School. Everyone connected with the hospital during the time that Miss Monk was matron is asked to contribute, however small the amount. Donations should be sent to the Secretary, Monk Memorial Committee, King's College Hospital, Denmark Hill, S.E.

### PASSING EVENTS AND TOPICS.

### New Hospital for Women, Euston Road.

Her Majesty the Queen, attended by the Countess of Minto, recently paid an informal visit to the New Hospital for Women, Euston Road. Her Majesty visited the out-patient department, and went through the main wards, the balconies, and the four small private wards of the hospital. The Queen gave great pleasure by saying a few words to each patient.

### The Pharmaceutical Register.

The report of the Registrar of the Pharmaceutical Society shows that the number of names on the Register of Chemists and Druggists is 16,799, an increase on the previous year of 169. *Per contra* there has been a heavy falling-off in the number of students.

### New Medical Officer at Stockport Union.

Dr. J. Howie Smith has been appointed medical officer of the Stockport Union Workhouse and Stepping Hill Hospital, in succession to the late Dr. Barker Bale, whose deputy he had been for fifteen years. Notwithstanding a letter from the Local Government Board inspector that such appointments should not be filled permanently during the war, when medical men were serving in the Army who might be candidates, Dr. Smith's election was passed by eighteen votes to twelve.

### Leicester Royal Infirmary.

Legacies Received.—The treasurer of the Leicester Royal Infirmary has received £200 from the estate of the late Mrs. E. L. Pochin, £200 from the estate of the late Miss E. A. Goddard, and £1,053 from the estate of the late Madame Brizio.

Legacies Notified.—Intimations of a legacy of £300 under the will of the late Mr. Joseph Buxton, and residuary bequests under the wills of the late Mr. G. L. Porter, Miss Elizabeth Slade, Mr. Wm. Lander, and Miss Emma Cooper are also reported.

### Stamping out Typhoid.

If further evidence were required of the value of anti-typhoid inoculation it is to be found in the Report of the Surgeon-General of the United States Army, which states that in a total mean strength of 98,649 men in 1914, only seven cases of typhoid occurred, and of these only two had received the complete course of vaccination. The report of the Surgeon-General states that the inoculation procedure now in use in the Navy is to inoculate on entry into the Service and every four years afterwards. As a result of this system, hospital provisions made for large numbers of typhoid cases have been appropriated to other uses.

### A Leicester Worthy.

A well-known Leicester practitioner of the old type, Alderman Charles Lakin, has just died. He qualified L.R.C.P. and L.M. Edin. in 1872, L.S.A. in 1873, L.A.H. (Dublin) in 1874, and L.F.P.S. (Glas.) in 1877. In 1885 he was elected to membership of the Leicester Town Council, and in 1892 an Alderman, an office he held at the time of his death. He was a member of the Sanitary and Asylum Committees, on which he rendered long and useful service. He was also chairman of the Infectious Diseases Hospital Sub-Committee, a position in which his professional knowledge was of great value to the borough. As hon. medical officer to the Wycliffe Society for the Blind his sympathetic nature rendered his service to the blind of very real value. Alderman Lakin was Mayor of the Borough of Leicester in 1903. He passed away at the Home Hospital, Leicester, most useful and admirable of institutions of its type.

### An Appeal to Smokers.

At the Home for Disabled Belgian Soldiers, 45 Courtfield Gardens, Kensington, S.W., one of the Homes maintained by the Wounded Allies Relief Committee, of Sardinia House, Kingsway, W.C., important work is being done to help the inmates to become self-supporting by teaching them the arts of basket-making and poker-work, and already many of the disabled men are turning out well-made baskets of various kinds and useful articles in poker-work, including boxes suitable for cards, counters, trinkets, and so on, made from wooden cigar-boxes. Gifts of these boxes will be gratefully received either at Sardinia House or at the Home. By this means it will be possible to devote a larger share of the profits to the direct benefit of the workers.

RATES OF SUBSCRIPTION: Twelve months, 6/6; Six months, 4/-; Three months, 2/-, post free to any address in the United Kingdom. Abroad, Twelve months, 8/8; Six months, 5/-; Three months, 2/6, post free.—Address The Subscription Dept., "THE HOSPITAL," The Hospital Building, 28/29 Southampton Street, Strand, London, W.C.

